# Tricuspid Valve Infective Endocarditis Seen on Point-of-Care Ultrasound: A Case Report

**DOI:** 10.7759/cureus.116185

**Published:** 2026-09-13

**Authors:** Hannah P Sterndorf, Isabella R Cheatwood, David C Pigott, Elissa Corsino, Blakely K Hudson, Mustafa I Ahmed, Luke R Bishop

**Affiliations:** 1 Emergency Medicine, University of Alabama at Birmingham Heersink School of Medicine, Birmingham, USA; 2 Emergency Medicine, University of Alabama at Birmingham, Birmingham, USA; 3 Cardiology, University of Alabama at Birmingham, Birmingham, USA

**Keywords:** iv drug-use, point of care echocardiography, point-of-care-ultrasound, tricuspid valve endocarditis, tricuspid vegetation

## Abstract

Intravenous drug use is a major risk factor for infective endocarditis (IE), particularly right-sided disease involving the tricuspid valve. Early diagnosis remains challenging as patients often present with nonspecific symptoms, while delays in recognition can result in severe complications including septic emboli, valvular dysfunction, and sepsis. Point-of-care ultrasound (POCUS) has emerged as a valuable bedside modality capable of rapidly identifying valvular abnormalities and expediting the diagnosis of IE in the emergency department. We present a case of tricuspid valve infective endocarditis in a patient with a history of intravenous drug use in whom cardiac POCUS identified a large tricuspid vegetation prior to comprehensive echocardiography. Subsequent evaluation demonstrated methicillin-resistant *Staphylococcus aureus* (MRSA) bacteremia with multiple septic embolic complications. The patient ultimately underwent percutaneous vegetation debulking in addition to intravenous antibiotic therapy. This case highlights the utility of bedside cardiac POCUS in facilitating rapid recognition, expedited specialty consultation, and timely management of infective endocarditis in high-risk patients presenting to the emergency department.

## Introduction

Infective endocarditis (IE) is a life-threatening infection of the endocardial surface of the heart associated with substantial morbidity and mortality despite advances in antimicrobial therapy and cardiac imaging [1,2]. The incidence of IE has increased in recent years, partially driven by rising rates of intravenous drug use and invasive procedures [1,3]. Patients who inject drugs (PWID) represent a growing segment of the emergency department population, with an estimated 3.7 million PWID in the United States in 2018, representing approximately 1.5% of the adult population [4]. This population is at increased risk for multiple infectious complications including skin and soft tissue infections, bacteremia, HIV, hepatitis C, septic arthritis, pneumonia, empyema, and infective endocarditis [4]. IE in PWID tends to occur in younger patients, demonstrates a female predominance in younger patients, and is associated with high rates of recurrent infection [5,6]. Right-sided infective endocarditis, particularly involving the tricuspid valve, is most common in this population, with methicillin-resistant *Staphylococcus aureus* (MRSA) and *S. aureus* serving as predominant causative organisms [6-9]. MRSA-associated endocarditis is particularly concerning due to its aggressive clinical course, embolic complications, prolonged intravenous antibiotic requirements, and increasing antimicrobial resistance, making early recognition and treatment critical [9,10]. Standard treatment typically involves prolonged intravenous antibiotic therapy with vancomycin, while severe cases may require surgical or percutaneous endovascular intervention [9].

Diagnosis of IE remains challenging because patients often present with nonspecific symptoms. Echocardiography remains central to diagnosis and is incorporated into the 2023 update to the modified Duke criteria, though access to comprehensive transthoracic or transesophageal echocardiography may be delayed in emergency or resource-limited settings [11-13]. Additionally, the sensitivity of transthoracic echocardiography (TTE) for detecting vegetations remains limited, and absence of visible vegetations on TTE does not exclude IE [9]. Large vegetations greater than 20 mm are associated with significantly increased mortality and frequently produce severe tricuspid regurgitation leading to right-sided heart failure [9,14].

Point-of-care ultrasound (POCUS) has emerged as a valuable bedside modality for rapid cardiac assessment in emergency and critical care settings. Focused cardiac ultrasound can identify valvular abnormalities (including regurgitation), vegetations, and associated hemodynamic complications while expediting clinical decision-making. Prior evidence has demonstrated the utility of cardiac POCUS in facilitating earlier recognition of infective endocarditis prior to confirmatory echocardiography [13,15]. Earlier identification through bedside ultrasound may allow for faster blood culture acquisition, antibiotic initiation, specialty consultation, and escalation of care.

## Case presentation

A 33-year-old woman with a past medical history of intravenous (IV) drug use and prior femoral shaft and rib fractures following a motor vehicle collision presented to the emergency department with complaints of bilateral lower extremity pain and swelling and was now unable to walk. She endorsed shortness of breath but denied fever or chills. The patient also reported petechiae on her skin for the past week. She denied chest pain, nausea, vomiting, dysuria, constipation, and diarrhea, and she denied any known history of heart disease or heart failure.

On initial presentation, she was afebrile and tachycardic to 115 beats per minute, with normal blood pressure. The patient appeared cachectic, chronically ill, and in moderate distress. Physical examination was significant for bilateral lower extremity pitting edema extending to the hips, as well as petechiae involving both the upper and lower extremities.

Laboratory studies revealed a white blood cell count (WBC) of 11.19×10^3^/cmm, lactic acid of 3.3 mmol/L, hemoglobin of 6.7 g/dL, platelet count of 134×10^3^/cmm, and B-type natriuretic peptide (BNP) of 269 pg/mL. Erythrocyte sedimentation rate (ESR) and C-reactive protein (CRP) were both elevated at 32 mm/h and 88.42 mg/L, respectively (Table 1).

**Table 1:** Laboratory Studies with Patient's Values and Reference Range WBC: White blood cells; BNP: B-type natriuretic peptide; ESR: erythrocyte sedimentation rate; CRP: C-reactive protein.

Laboratory Study	Patient’s Values	Reference Range
WBC (×10^3 ^cmm)	11.19	4.00-11.00
Lactic Acid (mmol/L)	3.3	0.5-2.2
Hemoglobin (g/dL)	6.7	11.3-15.2
Platelet (×10^3^/cmm)	134	150.0-400.0
BNP (pg/mL)	269	0.0-100.0
ESR (mm/h)	32	0-20
CRP (mg/L)	88.42	0.00-10.90

Cardiac POCUS was performed by the emergency department (ED) physician, and the apical four-chamber view demonstrated a large mobile vegetation attached to the tricuspid valve with moderate tricuspid regurgitation (TR) adjacent to the interatrial septum (Figures 1, 2). Left ventricular ejection fraction (LVEF) was normal. A small pericardial effusion was also noted.

**Figure 1:**
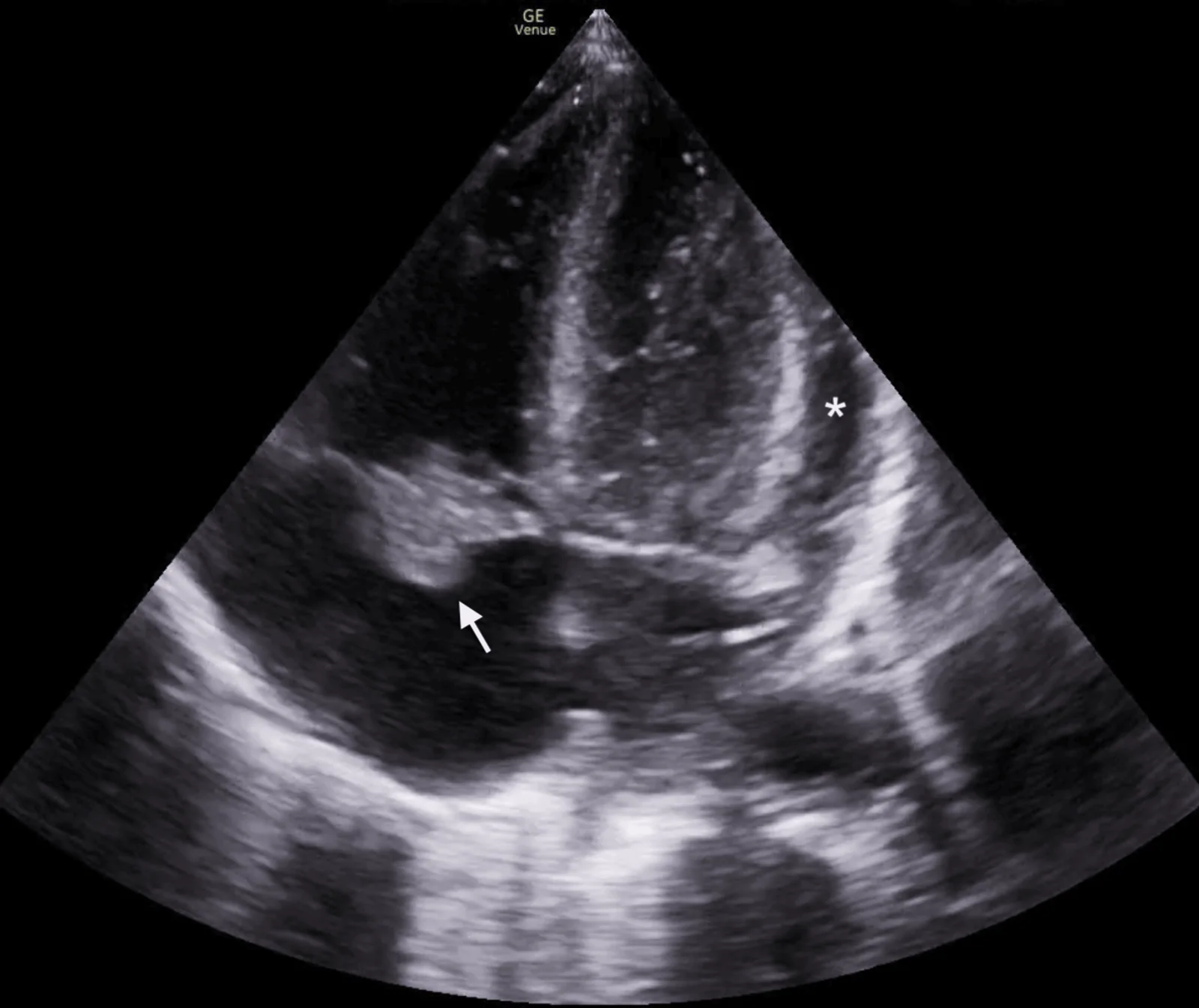
Ultrasound echo image showing tricuspid vegetation (arrow) and small pericardial effusion (*)

**Figure 2:**
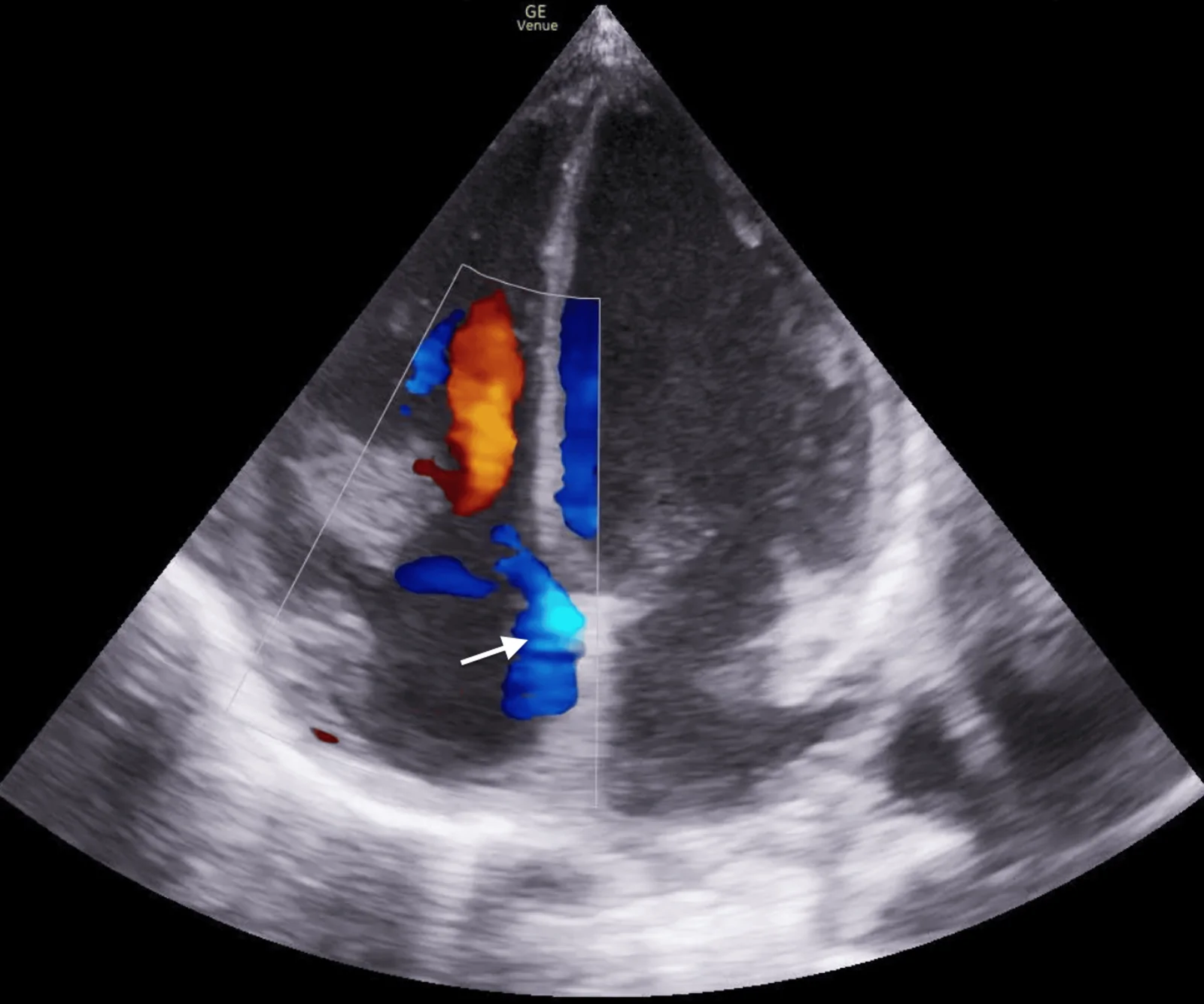
Ultrasound echo image showing tricuspid regurgitation (arrow) next to tricuspid valve vegetation

The patient’s presentation and POCUS findings were highly suggestive of tricuspid valve infective endocarditis (TVIE) in the setting of IV drug use. Three sets of blood cultures were obtained, and the patient was started on IV vancomycin and cefepime. Chest radiograph demonstrated multiple bilateral asymmetric airspace opacities with a small pleural effusion (Figure 3). Chest CT further demonstrated multiple pulmonary cavitary lesions consistent with septic emboli.

**Figure 3:**
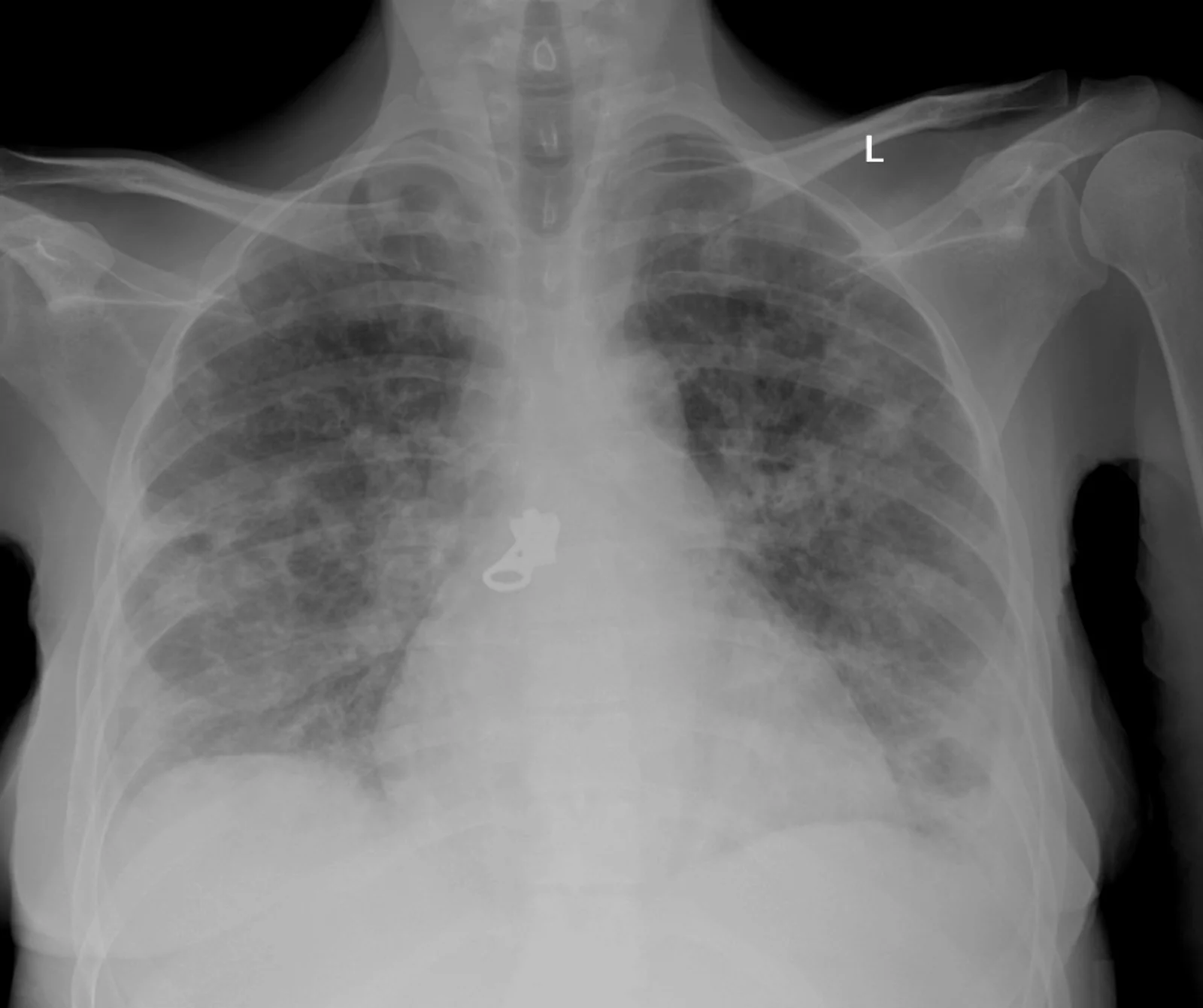
Chest X-ray showing bilateral, multiple asymmetrical airspace opacities with a small left sided pleural effusion

A urine drug screen obtained in the emergency department was positive for cocaine, heroin, fentanyl, opiates, and oxycodone. Cardiology was consulted, and the patient was admitted to the hospital for further management.

All three blood culture sets obtained in the emergency department were positive for MRSA, while sputum cultures grew *Pseudomonas aeruginosa*. During admission, TTE demonstrated findings similar to those seen on ED POCUS, including a tricuspid vegetation measuring greater than 2 cm in diameter. The patient subsequently underwent transesophageal echocardiography (TEE), which demonstrated a large vegetation on the posterior leaflet of the tricuspid valve with severe TR.

Cardiovascular surgery was consulted for possible operative intervention; however, due to the patient’s history of leaving against medical advice (AMA) and refusing care, she was not considered an ideal surgical candidate. Subsequently, she underwent successful percutaneous vegetation debulking using the AngioVac device (AngioDynamics, Latham, NY, USA).

During admission, the patient was also followed by infectious disease specialists and remained on IV vancomycin and cefepime. Her hospital course was complicated by multiple embolic phenomena, including cavitary and noncavitary pulmonary lesions with bilateral pleural effusions, renal infarcts, and a left iliac crest abscess.

## Discussion

This case highlights the need for POCUS in the ED as shown by the aggressive nature of TVIE and its potential for widespread embolic complications. PWID represent a growing proportion of emergency department presentations in the United States [4]. Right-sided IE, particularly involving the tricuspid valve, is strongly associated with intravenous drug use due to repeated inoculation of skin flora and contaminants into the venous circulation [7,16]. *S. aureus*, especially MRSA, remains one of the most common causative organisms in this patient population [7,10].

Our patient developed further complications commonly attributed to TVIE, including septic pulmonary emboli, cavitary lung lesions, renal infarcts, pleural effusions, and abscess formation. Large vegetations and persistent bacteremia are associated with worse clinical outcomes and increased mortality [14]. Although IE may initially present with nonspecific symptoms, this case demonstrates how bedside cardiac ultrasound can facilitate early identification of significant valvular pathology in high-risk patients. In this case, recognizing important past medical history and a thorough physical exam allowed for prompt bedside ultrasound and faster treatment.

The use of cardiac POCUS in the emergency department continues to expand and has become increasingly valuable in identifying life-threatening cardiac pathology. TTE and TEE remain the diagnostic standards for infective endocarditis, but ED POCUS is a necessary and significant way to reduce future risks. In this case, bedside POCUS identified a large tricuspid vegetation and associated tricuspid regurgitation prior to formal echocardiography, expediting further diagnostic evaluation, antibiotic administration, specialty consultation, and definitive management. As access to bedside ultrasound and physician familiarity with cardiac POCUS continue to improve, its role in reducing delays in diagnosis and management of infective endocarditis may continue to expand.

The management of severe TVIE frequently requires a multidisciplinary approach involving emergency medicine, cardiology, infectious disease, and cardiovascular surgery teams. Surgical intervention is often considered in patients with large vegetations, recurrent septic emboli, persistent bacteremia, or severe valvular dysfunction [8]. However, patients with substance use disorders may present unique barriers to traditional surgical management due to social and medical complexities, including recurrent AMA discharge and nonadherence to care [17]. This case additionally demonstrates the potential role of percutaneous vegetation debulking with the AngioVac system as a less invasive therapeutic option in selecting high-risk patients who may not be ideal surgical candidates [18].

## Conclusions

This case highlights the severe complications associated with tricuspid valve infective endocarditis in PWID, including septic pulmonary emboli and tricuspid regurgitation. As intravenous drug use continues to rise, emergency physicians are increasingly encountering patients at high risk for infective endocarditis, making early diagnosis essential to reducing morbidity and mortality.

This case also demonstrates the growing utility of POCUS in the emergency department for the rapid identification of significant cardiac pathology. In this patient, bedside cardiac POCUS allowed for early visualization of a large tricuspid vegetation prior to formal echocardiography, thereby expediting diagnostic evaluation, initiation of antibiotics, and specialty consultation. Existing literature and this case report specifically support the expanding role of POCUS as a valuable adjunct in the early recognition and management of infective endocarditis, particularly in high-risk patient populations such as PWID.
